# Effects of heat stress on laying performance, blood hormones and biochemical indices, egg quality, and intestinal digestive enzymes in laying ducks

**DOI:** 10.1016/j.psj.2026.107617

**Published:** 2026-08-15

**Authors:** Yuji Sun, Guomin Zhang, Xingda Yang, Rihong Guo, Fang Chen, Shijia Ying

**Affiliations:** a College of Animal Science and Technology, Nanjing Agricultural University, Nanjing 210095, China; b Institute of Animal Science, Jiangsu Academy of Agricultural Sciences, Nanjing 210014, China; c School of Ecology and Applied Meteorology, Nanjing University of Information Science and Technology, Nanjing 210044, China

**Keywords:** High temperature stress, Egg production of laying ducks, Reproductive hormones, Egg quality, Digestive enzyme activity

## Abstract

Chronic heat stress poses a major challenge to duck production by impairing reproductive performance and physiological homeostasis. This study investigated the effects of chronic heat stress on laying performance, reproductive physiology, blood biochemical indices, egg quality, intestinal digestive enzyme activities, and hepatic oxidative status in Gaoyou sheldrakes. Sixty 16-week-old ducks were randomly assigned to either a thermoneutral group (21 ± 2°C) or a heat stress group (35 ± 2°C, 24 h/day), with heat exposure initiated at 24 weeks of age. Compared with the thermoneutral group, chronic heat stress significantly reduced feed intake, laying rate, egg weight, ovarian development, serum estradiol and progesterone concentrations, and egg quality, while markedly altering blood biochemical parameters indicative of impaired nutrient metabolism and liver function. Heat stress also significantly decreased the activities of digestive enzymes in the small intestine and disrupted hepatic oxidative homeostasis, as evidenced by increased HSP70 and MDA levels together with reduced antioxidant enzyme activities. These findings demonstrate that chronic heat stress impairs laying performance through coordinated alterations in intestinal digestion, hepatic oxidative balance, nutrient metabolism, and reproductive endocrine function, providing new insights into the physiological mechanisms underlying heat stress-induced reproductive dysfunction in laying ducks and supporting the development of effective mitigation strategies for duck production under high-temperature conditions.

## Introduction

Global warming has increased the frequency, intensity, and duration of heat waves worldwide, making heat stress one of the major challenges threatening the sustainable development of the poultry industry (Oluwagbenga and Fraley, 2023). Poultry are particularly susceptible to elevated ambient temperatures because they lack sweat glands, possess a high metabolic rate, and are covered with dense feathers, all of which limit their ability to dissipate excess body heat. When environmental temperatures exceed approximately 28°C, birds exhibit physiological and behavioral responses associated with heat stress (Zhang et al., 2025; Zhao et al., 2024). Egg production is among the most heat-sensitive economic traits in commercial poultry. Previous studies have demonstrated that environmental temperatures above 29°C significantly reduce laying rate, egg weight, and feed intake in laying hens (Wang et al., 2025; Yunitasari et al., 2023). Similarly, exposure of laying ducks to high temperatures (32–38°C) has been reported to decrease feed intake, egg production, eggshell quality, and egg weight while increasing the incidence of broken eggs (Liu et al., 2023; Li et al., 2023).

Beyond impairing productive performance, heat stress induces a series of physiological and metabolic disturbances in poultry. In chickens, prolonged heat stress disrupts endocrine homeostasis, alters blood biochemical profiles, suppresses intestinal digestive and absorptive functions, impairs intestinal barrier integrity, and induces immune dysfunction, ultimately leading to reduced growth and reproductive performance (Sumanu et al., 2023; Xiao et al., 2023; Yuan et al., 2022; Tan et al., 2022). Heat stress has also been shown to impair ovarian function by reducing circulating reproductive hormone concentrations, inhibiting follicular development, and decreasing ovulation efficiency. Furthermore, decreased activities of intestinal digestive enzymes, including amylase and lipase, may reduce nutrient utilization and subsequently compromise egg formation and deposition (Ahmad et al., 2024; Lu et al., 2025; Song et al., 2018). These findings indicate that the detrimental effects of heat stress involve coordinated alterations in reproductive endocrinology, metabolism, and digestive physiology.

Although considerable progress has been made in elucidating the physiological mechanisms of heat stress in chickens, comparable studies in laying ducks remain limited. In particular, previous studies have mainly focused on individual physiological responses, whereas comprehensive evaluations integrating laying performance, reproductive endocrine function, blood biochemical metabolism, egg quality, and intestinal digestive function under chronic heat stress are scarce. Consequently, the physiological relationships among these systems and their collective contribution to heat stress-induced declines in laying performance in ducks remain poorly understood.

Therefore, the present study established a controlled environmental heat stress model to simulate the natural high-temperature conditions encountered during summer. Laying ducks were exposed to either thermoneutral or continuous high-temperature conditions, and changes in laying performance, reproductive hormone concentrations, blood biochemical indices, egg quality, and intestinal digestive enzyme activities were comprehensively evaluated. By integrating these physiological indicators, this study aimed to clarify the mechanisms underlying heat stress-induced impairment of laying performance in ducks and to provide a theoretical basis for developing effective heat stress mitigation strategies and improving the productivity and welfare of laying ducks under high-temperature conditions.

## Materials and methods

### Ethics approval

All animal protocols and experiments were conducted in accordance with the principles and specific guidelines presented in the Regulations for the Administration of Affairs Concerning Experimental Animals (Decree No. 63 of the Jiangsu Academy of Agricultural Sciences, July 8, 2014).

### Animals and experimental design

Sixty healthy 108-day-old (16-week-old) Gaoyou sheldrakes with uniform body weight were selected as the experimental subjects, all purchased from the National Gaoyou Sheldrakes Base in Gaoyou City, Jiangsu Province. A completely randomized design was adopted, and the ducks were randomly assigned to two groups with three replicates per group. Each group was housed in a 12 m² environmental chamber and evenly distributed among three pens, with 10 ducks per pen. They were raised in a temperature-controlled cabin with a temperature of 21°C and a relative humidity of 60%. The number of eggs laid and the amount of leftover feed in each group were recorded daily to calculate the daily laying rate, average egg weight, daily average feed intake, and feed-to-egg ratio. At 168 days of age (24 weeks), 6 ducks from each group were randomly slaughtered. The relative humidity remained at 60%, while the temperature of the heat stress group was continuously maintained at 35°C (24 h/day) throughout the experimental period. Two treatments were set: the normal temperature group (21 ± 2°C) and the high temperature group (35 ± 2°C). At 273 days of age (39 weeks), 9 ducks from each group were randomly selected and slaughtered. Body weight was measured immediately before slaughter, and the body weight data are presented in Supplementary Material (Fig. S1). During the experimental period, mortality was 2 ducks in the control group and 7 ducks in the heat stress group.

### Feeding management

The experiment was conducted from November 2024 to April 2025 in the artificial climate-controlled room of the Liuhe Experimental Poultry Farm of Jiangsu Academy of Agricultural Sciences. The environmental temperature and relative humidity were automatically adjusted by an intelligent control system according to the set parameters (temperature accuracy ±1°C, humidity accuracy ±5%). The recorded temperature, relative humidity, and temperature–humidity index (THI) throughout the experimental period are presented in Supplementary Material (Fig. S2). The lighting program followed the traditional 16:8 parameter mode for egg-laying ducks, with free-range floor housing, free feeding, and free drinking.

The experimental feed was standard commercial feed for egg-laying ducks, meeting the requirements of the Feed Hygiene Standard (AQSIQ, 2017) GB13078-2017 and the nutritional requirements of the Ministry of Agriculture and Rural Affairs' Egg-Laying Duck Nutrient Requirements (SAC, 2021) GB/T 41189-2021. The feed formulation and nutrient composition are provided in the Supplementary Material (Table S1). The feed was provided once daily at 14:00, and the daily feed amount and leftover feed were recorded for subsequent index calculations.

### Egg quality collection and measurement

The whole eggs were collected daily at 14:30, numbered individually, weighed using an electronic balance, and recorded as the daily egg weight. From 25 weeks to 38 weeks of age, 10 eggs with normal appearance were randomly collected from each group every three days (Monday to Wednesday) for egg quality determination. The collected eggs were gently wiped with a soft cloth to remove surface dirt, labeled with group and date, and all egg quality indicators were determined within 24 hours.

### Serum samples collection

The ducks were sacrificed by jugular vein bleeding, and whole blood was collected using non-EDTA blood collection tubes (5 ml). Blood samples were collected between 09:00 and 11:00 on the day of slaughter. To minimize physiological variation, all ducks were sampled at a comparable oviposition time and reproductive physiological stage. The blood samples were placed in an insulated box with ice packs and centrifuged at 4°C and 2500 r/min for 15 minutes on the day of collection using a low-temperature centrifuge. The upper serum layer was carefully aspirated with a pipette and aliquoted into 1.5 ml sterile centrifuge tubes, immediately placed in a −20°C refrigerator for storage, and used for hormone and biochemical index determination.

### Follicle counting and intestinal sample collection

After slaughter, the number of hierarchical follicles was counted, and the maximum outer diameter of each hierarchical follicle, including the follicular theca, was measured using a digital vernier caliper. Intestinal sample collection: The duodenum (from the pylorus to the pancreatic duct), jejunum (from the pancreatic duct to the yolk sac), ileum (from the yolk sac to the ileocecal ligament), and cecum were rapidly separated. Intestinal mucosa collection: About 3 cm of the middle section of each intestinal segment was selected, gently rinsed with pre-cooled physiological saline to remove intestinal contents. Then, the intestinal segments were longitudinally cut open, and the intestinal mucosa was scraped with a slide and placed in a cryotube, immediately immersed in liquid nitrogen for rapid freezing, and then transferred to a −80°C ultra-low temperature refrigerator for storage, for the determination of digestive enzyme activity. Intestinal content collection: About 1 g of the middle section contents of each intestinal segment was taken, placed in a cryotube, rapidly frozen in liquid nitrogen, and stored at −80°C for the analysis of content enzyme activity.

### Sample determination

Reproductive hormones: The concentrations of estradiol-17β(E2) and progesterone (P4) in serum were determined by competitive enzyme-linked immunosorbent assay (ELISA).

Blood biochemical index measurement: Serum total protein (TP, biuret method), albumin (ALB, bromocresol green method), blood glucose (GLU, glucose oxidase method), lactate dehydrogenase (LDH, rate method), total cholesterol (TC), low-density lipoprotein cholesterol (LDL-C), high-density lipoprotein cholesterol (HDL-C), triglycerides (TG), uric acid (UA), creatinine (Cr), alanine aminotransferase (ALT, rate method), and aspartate aminotransferase (AST, rate method) were measured using an automatic biochemical analyzer.

Egg quality measurement: Egg length and width were measured using a digital vernier caliper, and the egg shape index was calculated as the ratio of egg width to egg length. Eggshell strength was measured using an eggshell strength tester. Yolk weight, yolk color, albumen height, and Haugh unit were determined using an automatic egg quality analyzer.

Intestinal digestive enzyme activity measurement: Approximately 0.1 g of intestinal mucosa was weighed, and nine volumes of ice-cold physiological saline were added to prepare a 10% (w/v) tissue homogenate. The tissue was homogenized in an ice bath using a tissue homogenizer. The homogenate was centrifuged at 12,000 r/min for 10 min at 4°C using a refrigerated centrifuge, and the resulting supernatant was collected for enzyme activity determination. Total protein concentration was determined prior to enzyme assays, and all digestive enzyme activities were normalized to total protein content and expressed as U/mg protein.

The activities of amylase, lipase, protease, sucrase, and maltase were determined using commercially available assay kits obtained from Nanjing Jiancheng Bioengineering Institute (Nanjing, China), including total protein (Cat. No. A045-2-3), amylase (Cat. No. C016-1-2), lipase (Cat. No. A054-2-1), protease (Cat. No. A082-2), alkaline phosphatase (Cat. No. A059-2-1), sucrase (Cat. No. A082-2-1), and maltase (Cat. No. A082-3-1). Soluble starch, a triglyceride substrate, casein, sucrose, and maltose were used as the reaction substrates for amylase, lipase, protease, alkaline phosphatase , sucrase, and maltase assays, respectively. Enzyme activities were calculated according to the equations provided in the manufacturer's protocols and expressed as U/mg protein. Enzyme activities were calculated according to the equations provided by the manufacturer and normalized to protein concentration.

Determination of hepatic heat stress and oxidative stress biomarkers: Approximately 0.10 g of liver tissue was homogenized with nine volumes of ice-cold physiological saline to prepare a 10% (w/v) homogenate. The homogenate was centrifuged at 12,000 r/min for 10 min at 4°C, and the supernatant was collected for subsequent analyses. The concentrations of heat shock protein 70 (HSP70, Cat. No. H264-2-2), malondialdehyde (MDA, Cat. No. A003-1-2), and carnitine O-octanoyltransferase (CROT, Cat. No. H205-1-2), together with the activities of superoxide dismutase (SOD, Cat. No. A001-3-2), catalase (CAT, Cat. No. A007-1-1), and glutathione peroxidase (GSH-Px, Cat. No. A005-2-1), were determined using commercially available assay kits (Nanjing Jiancheng Bioengineering Institute, Nanjing, China) according to the manufacturer's instructions. Enzyme activities were normalized to total protein concentration and expressed as U/mg protein, whereas MDA, HSP70, and CROT concentrations were expressed as nmol/mg protein, ng/mg protein, and ng/mg protein, respectively.

### Statistical analysis

Statistical analyses were conducted using IBM SPSS Statistics. Homogeneity of variances was assessed using Levene’s test before comparisons between the two groups were performed with an independent-samples t-test. Hedges’g was calculated as a measure of effect size. Results are expressed as the mean ± SEM. A two-tailed *P* value < 0.05 was considered statistically significant, whereas *P* > 0.05 indicated no statistically significant difference between groups. Fig.s were prepared using GraphPad Prism 9.

## Results

### Effect of high temperature on laying performance of ducks

**Fig. 1:**
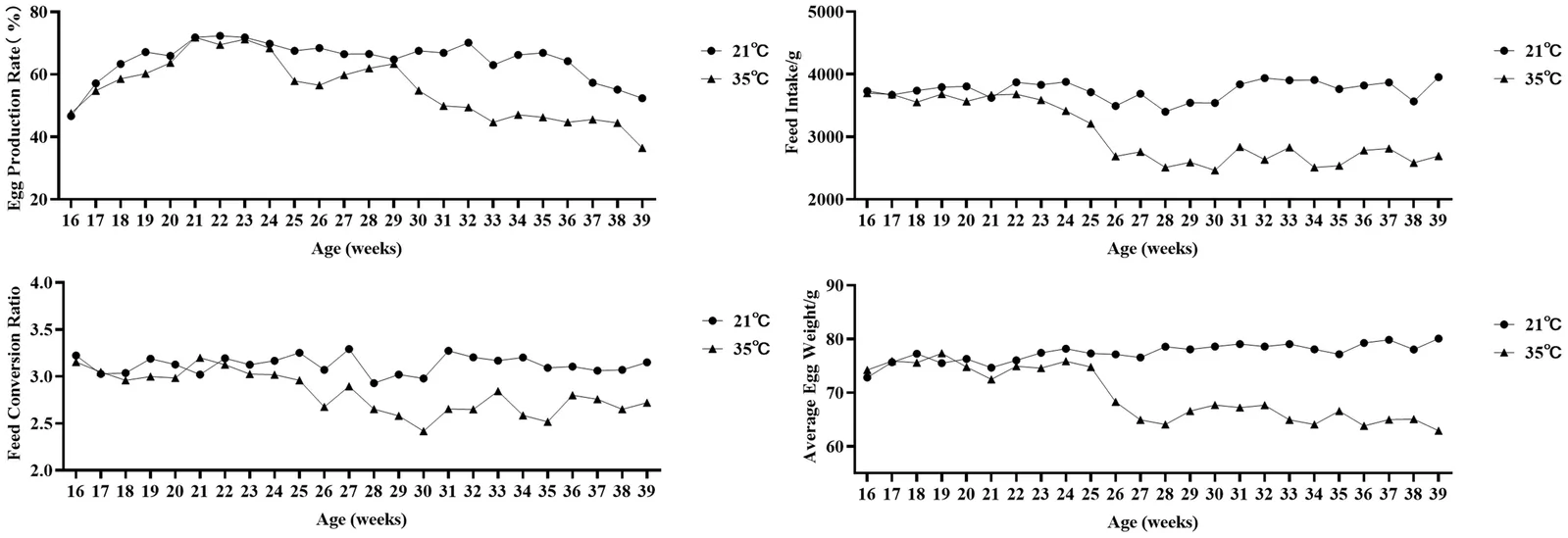
Curves of laying rate, feed intake, FCR, and average egg weight in laying ducks exposed to high temperature.

Before the temperature increase, no significant differences were observed in laying rate, feed intake, feed conversion ratio, or average egg weight between the 21°C and 35°C groups. During 25–26 weeks of age, ducks exposed to 35°C began to exhibit reductions in feed intake and average egg weight compared with the 21°C group. During 27–30 weeks of age, the decline in laying performance became more evident, with a progressive decrease in laying rate accompanied by an increase in feed conversion ratio. From 31 to 38 weeks of age, the adverse effects of prolonged heat stress became more pronounced, as the laying rate was significantly lower than that of the 21°C group and feed intake and average egg weight remained consistently lower, whereas the feed conversion ratio remained higher (Fig. 1).

### Effect of high temperature on reproductive hormones and follicular development

**Fig. 2:**
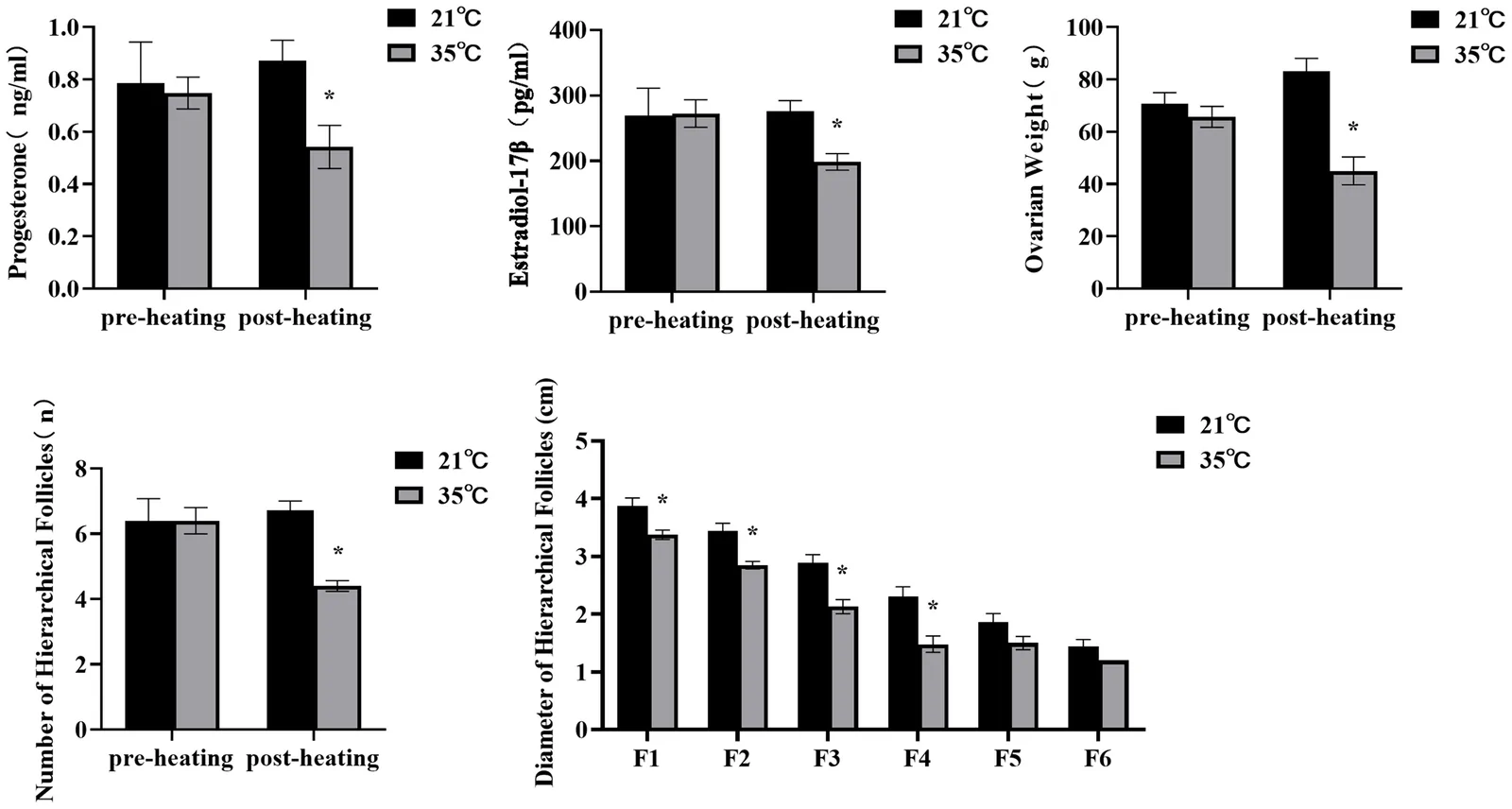
Effects of high temperature exposure on reproductive hormone levels, ovary weight, and the number and diameter of hierarchical follicles in Gaoyou sheldrakes. Data are presented as mean ± SEM (n = 9). An asterisk (*) indicates a significant difference between groups (*P* < 0.05); no asterisk indicates no significant difference (*P* > 0.05).

Before the temperature increase, no significant differences were observed between the 21°C and 35°C groups in estradiol-17β (E2), progesterone (P4), ovarian weight, the number of hierarchical follicles, or the diameters of hierarchical follicles (*P* > 0.05). After the temperature increase, serum E2 (*P* = 0.014) and P4 (*P* = 0.002) concentrations were significantly lower in the 35°C group than in the 21°C group. Ovarian weight (*P* = 0.001) and the number of hierarchical follicles (*P* = 0.001) were also significantly reduced. In addition, the diameters of hierarchical follicles F1 (*P* = 0.006), F2 (*P* = 0.001), F3 (*P* = 0.001), and F4 (*P* = 0.002) were significantly smaller in the 35°C group, whereas no significant differences were observed for F5 (*P* = 0.198) or F6 (*P* = 0.499) (Fig. 2). The corresponding effect sizes (Hedges’ g) and 95% confidence intervals are presented in Supplementary Material (Table S2).

### Effect of high temperature on blood biochemical indices

**Fig. 3:**
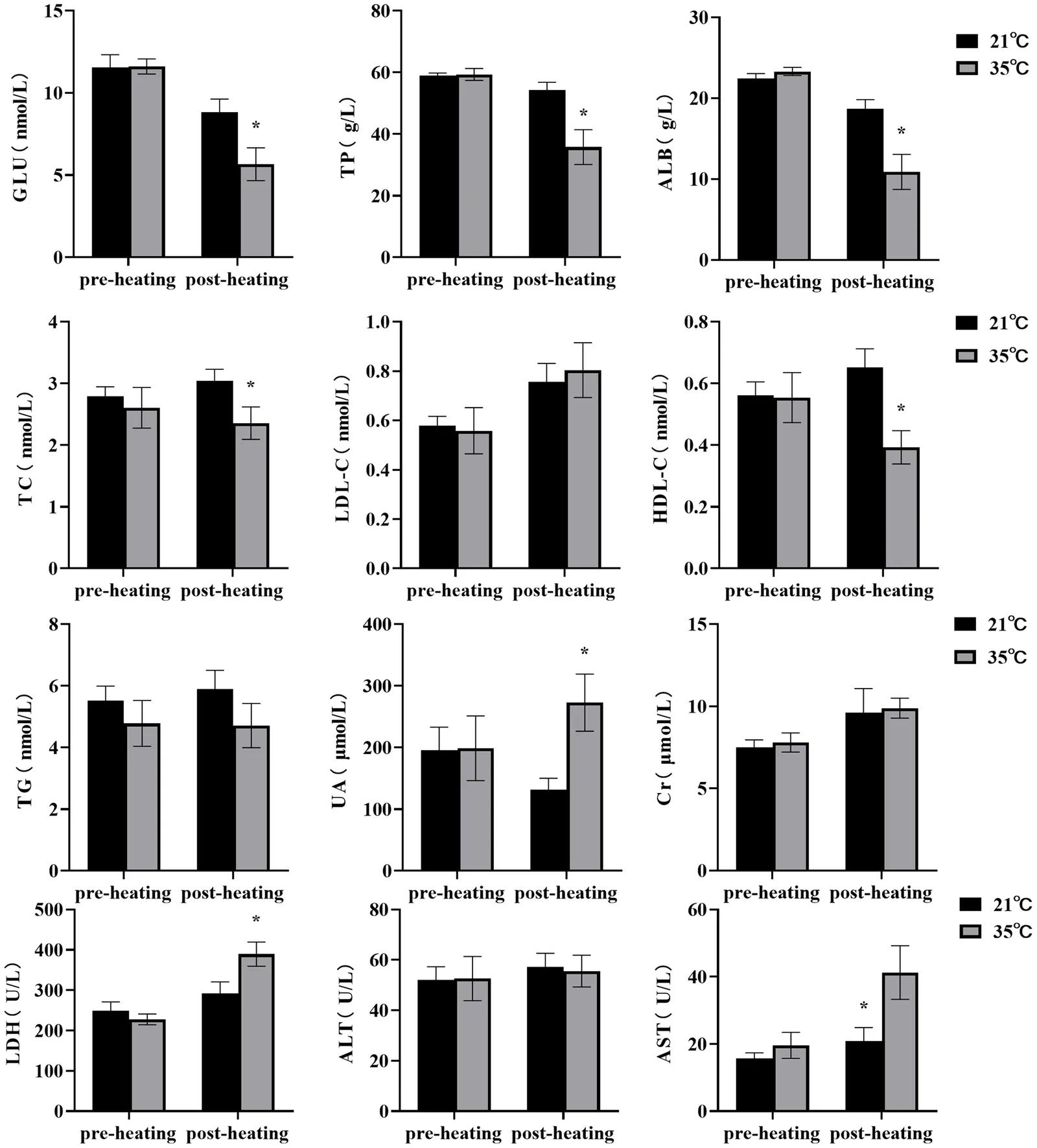
Effects of high-temperature exposure on blood biochemical indices. Data are presented as mean ± SEM (n = 9). An asterisk (*) indicates a significant difference between groups (*P* < 0.05); no asterisk indicates no significant difference (*P* > 0.05).

Before the temperature increase, no significant differences (*P* > 0.05) were observed in any measured blood biochemical parameter between the 21°C and 35°C groups. After the temperature increase, the concentrations of blood glucose (GLU, *P* = 0.001), total protein (TP, *P* = 0.011), albumin (ALB, *P* = 0.005), high-density lipoprotein cholesterol (HDL-C, *P* = 0.006), and total cholesterol (TC, *P* = 0.042) were significantly lower in the 35°C group than in the 21°C group. Conversely, uric acid (UA, *P* = 0.015), lactate dehydrogenase (LDH, *P* = 0.040), and aspartate aminotransferase (AST, *P* = 0.026) were significantly higher in the 35°C group. No significant differences were observed in triglycerides (TG, *P* = 0.219), low-density lipoprotein cholesterol (LDL-C, *P* = 0.741), alanine aminotransferase (ALT, *P* = 0.841), or creatinine (Cr, *P* = 0.870) (Fig. 3). Detailed effect sizes (Hedges' g) and 95% confidence intervals for all comparisons are provided in Supplementary Material (Table S2).

### Effect of high temperature on egg quality

**Fig. 4:**
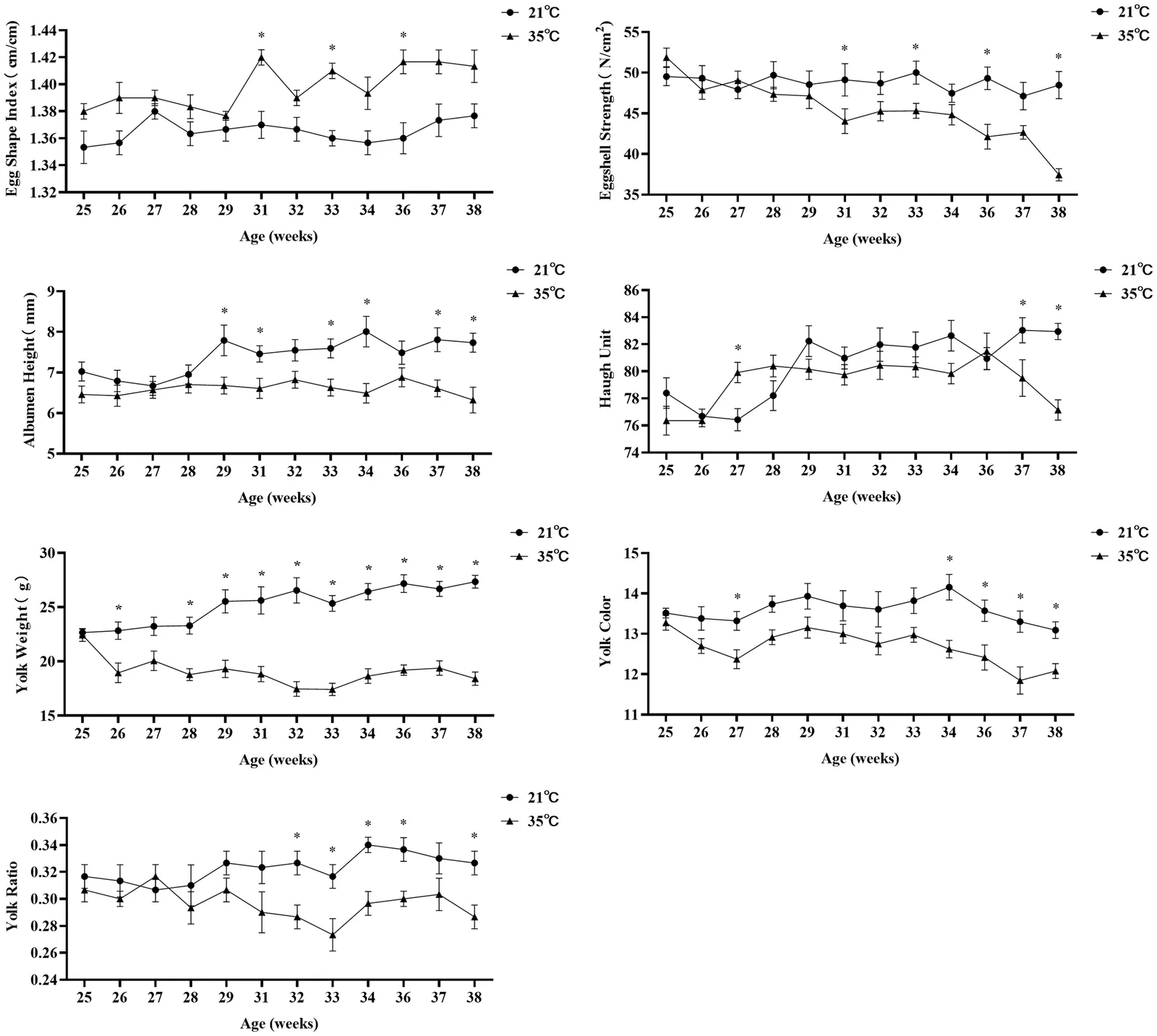
Effects of high temperature on egg quality-related indices.

During 25–28 weeks of age, no consistent differences were observed in egg quality parameters between the 21°C and 35°C groups, although significant differences were detected for some parameters at individual time points. During 29–32 weeks of age, the effects of prolonged heat stress became apparent. Compared with the 21°C group, ducks exposed to 35°C exhibited lower yolk weight and albumen height, with significant differences observed at several time points. During 33–38 weeks of age, the adverse effects of heat stress became more pronounced. Overall, eggshell strength, yolk weight, yolk color, yolk ratio, and albumen height were lower in the 35°C group than in the 21°C group, whereas the egg shape index tended to be higher. In contrast, the Haugh unit remained relatively stable throughout the experimental period, with no consistent differences between the two groups (Fig. 4).

### Effect of high temperature on intestinal digestive enzyme activities in ducks

**Fig. 5:**
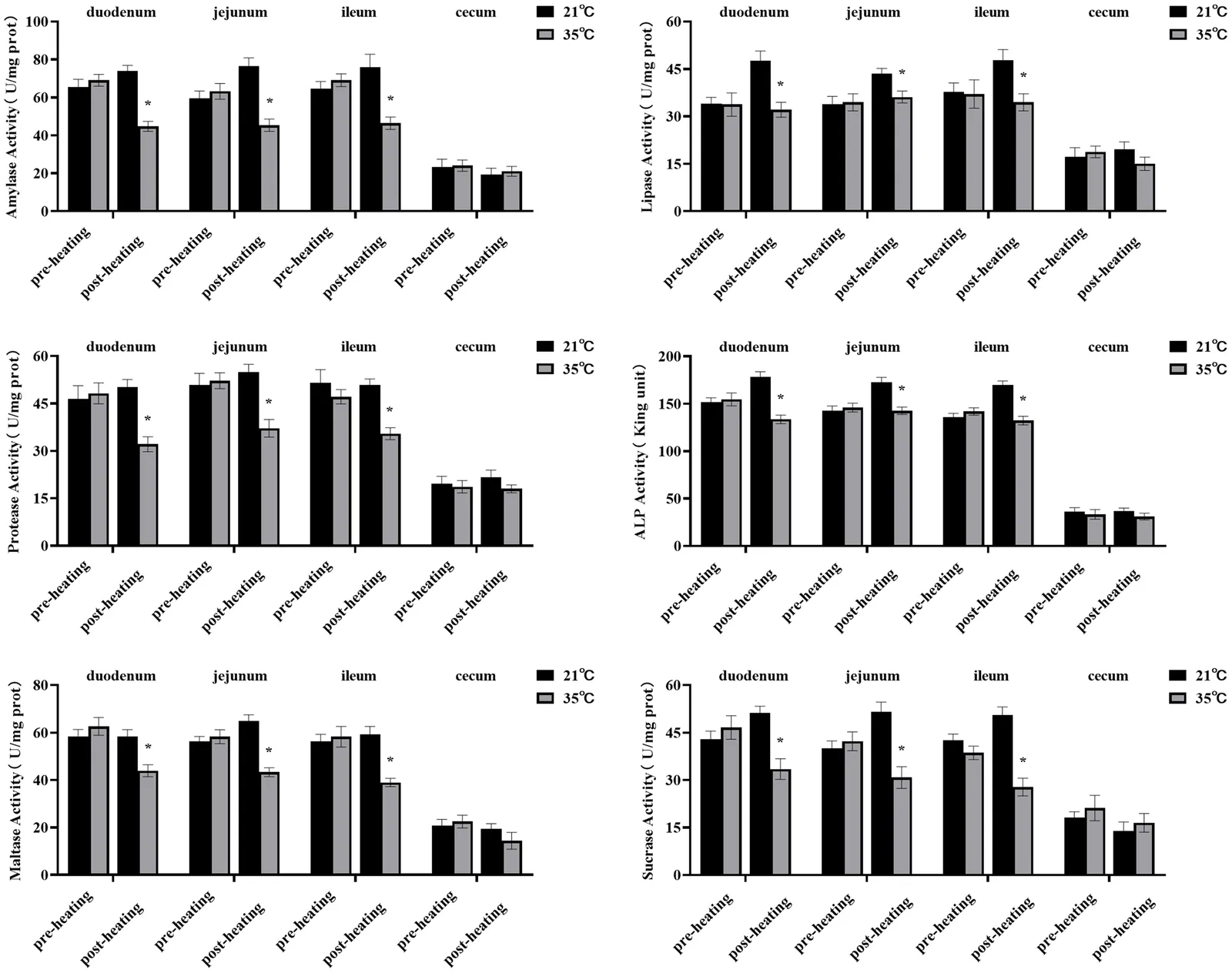
Effects of high temperature on intestinal digestive enzyme activities. Data are presented as mean ± SEM (n = 9). An asterisk (*) indicates a significant difference between groups (*P* < 0.05); no asterisk indicates no significant difference (*P* > 0.05).).

In the measurement of intestinal enzyme activities before and after the temperature increase, the overall enzyme activities in the duodenum, jejunum, and ileum were higher than those in the cecum. Before the temperature increase, there were no significant differences (*P* > 0.05) in intestinal digestive enzyme activities in the duodenum, jejunum, ileum, and cecum between the 21 °C and 35 °C groups. Compared with the 21°C group, ducks exposed to 35°C exhibited significantly lower activities of all six digestive enzymes in the duodenum, jejunum, and ileum, whereas no significant differences were observed in the cecum (Fig. 5). The exact p-values , detailed effect sizes (Hedges' g) and 95% confidence intervals for all comparisons are provided for all comparisons are provided in Supplementary Material (Table S2).

### Effect of high temperature on hepatic heat stress and oxidative stress biomarkers

**Fig. 6:**
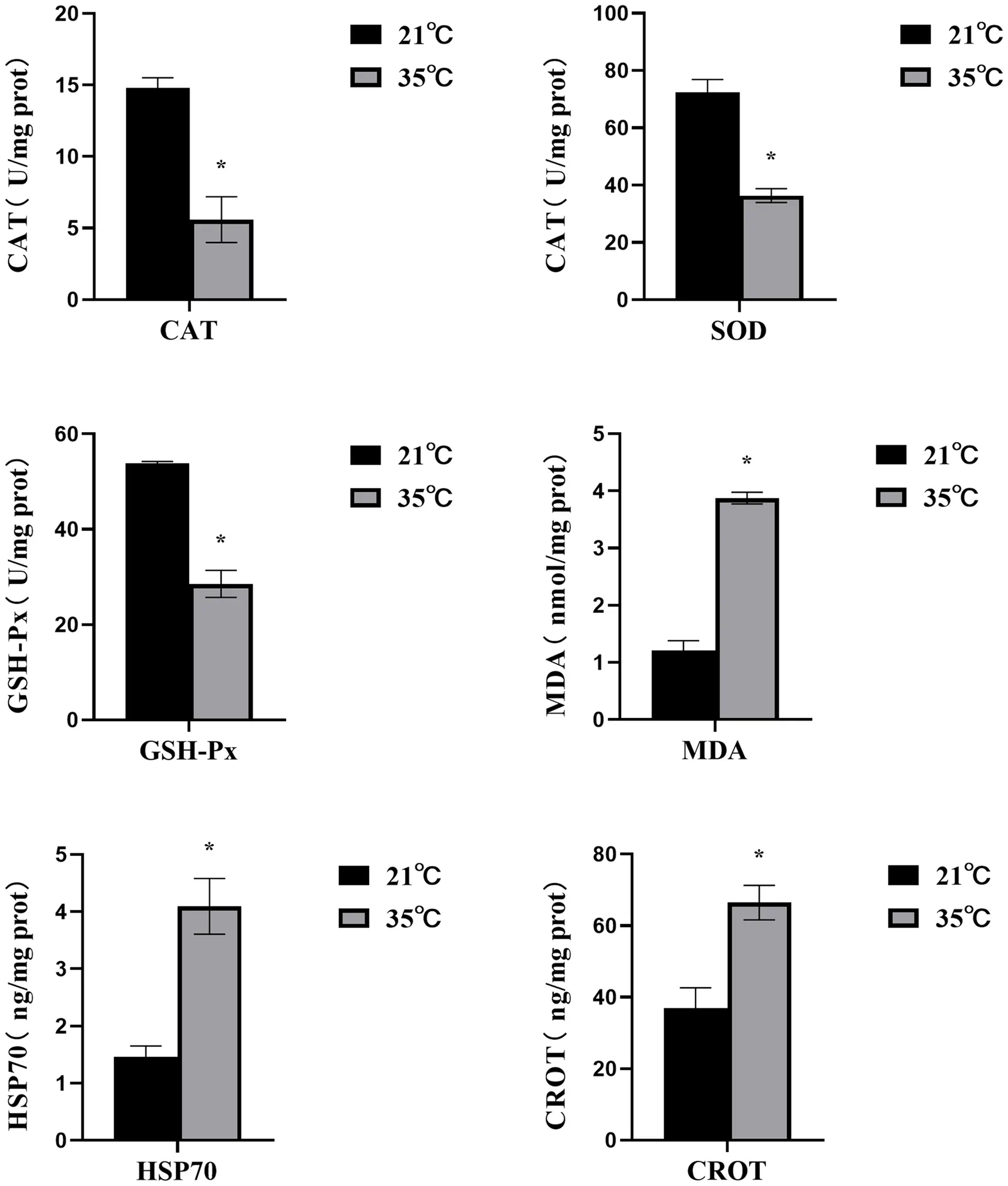
Effect of high temperature on hepatic heat stress and oxidative stress biomarkers. Data are presented as mean ± SEM (n = 9). An asterisk (*) indicates a significant difference between groups (*P* < 0.05); no asterisk indicates no significant difference (*P* > 0.05).).

Compared with the 21°C group, chronic heat stress significantly reduced the hepatic activities of catalase (CAT, *P* = 0.006), superoxide dismutase (SOD, *P* = 0.002), and glutathione peroxidase (GSH-Px, *P* = 0.001). In contrast, hepatic malondialdehyde (MDA, *P* = 0.001), heat shock protein 70 (HSP70, *P* = 0.007), and carnitine O-octanoyltransferase (CROT, *P* = 0.017) levels were significantly increased in the 35°C group (Fig. 6). These results indicate that prolonged exposure to high temperature induced a pronounced cellular heat stress response, impaired hepatic antioxidant defense capacity, enhanced lipid peroxidation, and altered hepatic fatty acid metabolism in laying ducks. Detailed effect sizes (Hedges' g) and 95% confidence intervals for all comparisons are provided in Supplementary Material (Table S2).

## Discussion

Chronic heat stress impaired laying performance through coordinated alterations in intestinal digestion, hepatic metabolism, oxidative homeostasis, and reproductive endocrine function. Rather than acting through a single physiological pathway, heat stress initiated a hierarchical cascade characterized by reduced feed intake, impaired digestive enzyme activities, decreased nutrient availability, disruption of reproductive hormone secretion, inhibition of follicular development, and ultimately reduced egg production and egg quality (Lara and Rostagno, 2013). This integrated physiological framework facilitates the interpretation of the phenotypic, biochemical, and molecular alterations observed in heat-stressed laying ducks.

High‑temperature exposure suppresses laying performance and deteriorates multiple egg quality traits via nutrient‑dependent and acid‑base‑mediated pathways. Reduced feed intake decreases the availability of nutrients required for hepatic metabolism, yolk precursor synthesis, and ovarian follicular development. Insufficient nutrient supply constrains the synthesis of vitellogenin and VLDLy, impairs yolk formation, and consequently decreases egg weight. Although calcium metabolism was not directly measured in the present study, previous studies have demonstrated that heat stress also induces respiratory alkalosis through excessive panting. The resulting increase in blood pH reduces ionized calcium availability and impairs calcium carbonate deposition in the shell gland, thereby weakening eggshell formation. In the current study, 35 °C‑stressed laying ducks exhibited lower eggshell strength, yolk weight, yolk color, yolk proportion and albumen height, accompanied by an elevated egg‑shape index relative to the 21 °C control group. With advancing experimental age, heat-stress ducks showed significant declines in laying rate, feed intake, feed‑to‑egg ratio and average egg weight. Faded yolk color under hyperthermia may be attributed to reduced carotenoid absorption or accelerated carotenoid catabolism. The progressive deterioration in laying performance observed during prolonged heat stress further indicates that the adverse effects of heat stress are cumulative rather than acute.These phenotypic changes are consistent with previous reports (Xiao et al., 2023; Ji et al., 2025; Vandana et al., 2021; Song et al., 2018; Liu et al., 2025; Cao et al., 2022), reflecting combined consequences of limited nutrient intake, disturbed calcium metabolism and oviduct mucosal injury (Saleh et al., 2019, Saleh et al., 2020; Garcia et al., 2019; Bagheri et al., 2019).

Reproductive suppression under heat stress is largely driven by HPA‑HPG axis crosstalk. Heat stress has been reported to activate the HPA axis, thereby elevating circulating corticosterone concentrations. Elevated corticosterone inhibits GnRH‑expressing neurons in the hypothalamus, which reduces pituitary synthesis and secretion of FSH and LH. The decreased gonadotropin levels further lower serum E₂ and P₄ concentrations, leading to retarded follicle development. Recent evidence further suggests that heat stress may suppress hypothalamic Kisspeptin‑GPR54 signaling, an upstream regulator of GnRH secretion, thereby exacerbating reproductive endocrine dysfunction. In agreement with this cascade, our results demonstrated that 35 °C treatment significantly decreased serum E₂ and P₄ levels and reduced both quantity and weight of dominant follicles (≥12 mm). Impaired development of pre‑ovulatory dominant follicles directly cuts down ovulation frequency, representing a core physiological cause for heat‑induced laying reduction in laying ducks (Idowu et al., 2026; Oladokun and Adewole, 2022).

The newly determined hepatic biomarkers further confirmed that chronic heat stress induced a classical cellular heat‑stress response. Increased hepatic HSP70 levels have been widely recognized as one of the most sensitive biomarkers of cellular heat stress in poultry Oladokun and Adewole (2022), thereby further supporting the successful establishment of the chronic heat stress model in the present study. Nevertheless, sustained heat stress disrupts oxidative homeostasis, evidenced by decreased activities of hepatic antioxidant enzymes including SOD, CAT and GPx (Oladokun and Adewole, 2022). Reduced antioxidant capacity provoked excessive ROS accumulation, which in turn increased hepatic MDA content and triggered hepatocyte injury, eventually disrupting whole‑body hepatic metabolic homeostasis. Increased hepatic CROT content may reflect an adaptive enhancement of hepatic fatty acid β‑oxidation in response to altered energy metabolism during chronic heat stress. The reduction in hepatic antioxidant enzyme activities together with increased MDA further supports that oxidative stress contributed to systemic metabolic dysfunction. Oxidative stress may also impair ovarian steroidogenesis by disrupting the function of granulosa and theca cells, while excessive ROS accumulation compromises follicular cell viability and accelerates follicular atresia, thereby providing an additional mechanistic link between hepatic oxidative damage, reproductive endocrine dysfunction, and reduced laying performance. These findings suggest that hepatic oxidative injury is not only a consequence of heat stress but also an important contributor to the decline in reproductive function observed in heat-stressed laying ducks (Mei et al., 2026; Saleh et al., 2022).

Intestinal dysfunction appears to be an important upstream event linking heat stress with systemic metabolic disturbances for altered blood biochemical profiles and subsequent production losses. Heat stress intestinal mucosal injury reduces duodenal, jejunal and ileal digestive‑enzyme activities. Amylase, lipase, and protease primarily reflect the digestion of carbohydrates, lipids, and proteins, respectively, whereas sucrase and maltase are brush‑border disaccharidases that indicate the digestive and absorptive capacity of intestinal epithelial cells. Therefore, the concurrent reduction in both luminal digestive enzymes and brush‑border disaccharidases suggests that chronic heat stress impaired not only nutrient digestion but also the functional integrity of the intestinal mucosa (Seo et al., 2024; Madkour et al., 2024). Suppressed digestive capacity impairs nutrient absorption, which leads to decreased circulating glucose, albumin and total cholesterol. Lower cholesterol limits precursor substrates for yolk synthesis, and such metabolic disturbance further aggravates the deterioration of egg production performance. The present study observed significant reductions in serum total protein, albumin, blood glucose and total cholesterol in the 35 °C group, while serum uric acid and aspartate aminotransferase were markedly increased compared with the control group. Although elevated uric acid is commonly considered indicative of enhanced protein catabolism, impaired renal urate excretion and dehydration associated with prolonged heat stress may also contribute to increased circulating uric acid concentrations. Elevated aspartate aminotransferase further indicates hepatic cell damage under high‑temperature conditions (Liu et al., 2021; Ma et al., 2014). Collectively, these biochemical alterations reflect the downstream metabolic outcomes originating from impaired intestinal digestion and absorption in heat‑stressed laying ducks (Saleh et al., 2023).

In summary, chronic heat stress impairs laying‑duck productivity and egg quality through interactive effects among intestinal digestion, hepatic oxidative metabolism and HPA‑HPG endocrine networks (Oluwagbenga and Fraley, 2023). From a practical perspective, improving dietary digestive efficiency through exogenous enzyme supplementation and alleviating reproductive endocrine dysfunction using antioxidant or phytoestrogen‑containing feed additives may represent promising nutritional strategies to mitigate heat stress in laying ducks (Mangan and Siwek, 2024). In addition, optimizing environmental cooling strategies to minimize prolonged thermal exposure should be considered a primary management practice. Future studies should further investigate different temperature gradients and durations of heat stress, together with the molecular regulation of HPA‑HPG crosstalk and intestinal‑liver‑reproductive axis interactions (Hossain et al., 2026). Collectively, the present study provides integrated physiological evidence linking intestinal dysfunction, hepatic oxidative stress, endocrine disruption, and impaired reproductive performance, thereby improving our understanding of the systemic mechanisms underlying chronic heat stress in laying ducks.

## Funding

This work was supported by the Jiangsu Provincial Key R&D Program-Modern Agriculture (grant no. BE2023339), and China Agriculture Research System (CARS): (CARS-41-38).

## Disclosures

The authors declare that they have no known competing financial interests or personal relationships that could have appeared to influence the work reported in the present study.

## Author Contributions

**Yuji Sun:** Data curation, Formal analysis, Writing – original draft. **Guomin Zhang:** Conceptualization, Methodology, Project administration. **Xingda Yang:** Investigation, Resources, Validation. **Rihong Guo:** Resources, Funding acquisition, Supervision. **Fang Chen:** Resources, Software, Supervision. **Shijia Ying:** Conceptualization, Writing – review & editing, Supervision.

## Disclosures

We would like to submit the enclosed manuscript entitled “Effects of Heat Stress on Production and Physiological Traits in Laying Ducks.”, which we wish to be considered for publication in ***Poultry Science.*** No conflict of interest exits in the submission of this manuscript, and manuscript is approved by all authors for publication. I would like to declare on behalf of my co-authors that the work described was original research that has not been published previously, and not under consideration for publication elsewhere, in whole or in part. All the authors listed have approved the manuscript that is enclosed.

## Footnotes

Supplementary material associated with this article can be found, in the online version, at doi:10.1016/j.psj.2026.107617.
