# Supplementary material for: Effects of heat stress on laying performance, blood hormones and biochemical indices, egg quality, and intestinal digestive enzymes in laying ducks
Source: Poult Sci. 2026 Aug 15;105(12):107617. doi: 10.1016/j.psj.2026.107617 (PMC13594849; doi:10.1016/j.psj.2026.107617)
Supplement: Supplementary file 1 [file mmc3.docx]

Supplementary Material

Title: Effects of heat stress on laying performance, blood hormones and biochemical indices, egg quality, and intestinal digestive enzymes in laying ducks.

Figure S1. Data of body weight slaughter (g).

Figure S2. The recorded temperature, relative humidity, and temperature–humidity index (THI) throughout the experimental period.

Table S1. Ingredient composition and calculated nutrient composition of the basal diet for laying ducks.

Table S2. The exact p-values , detailed effect sizes (Hedges' g) and 95% confidence intervals for all comparisons
