# Supplementary material for: Effects of heat stress on laying performance, blood hormones and biochemical indices, egg quality, and intestinal digestive enzymes in laying ducks
Source: Poult Sci. 2026 Aug 15;105(12):107617. doi: 10.1016/j.psj.2026.107617 (PMC13594849; doi:10.1016/j.psj.2026.107617)
Supplement: Supplementary file 2 [file mmc4.docx]

Table S1. Ingredient composition and calculated nutrient composition of the basal diet for laying ducks

| **Ingredient** | **%** |
| --- | --- |
| Corn | 59.50 |
| Soybean meal (43% CP) | 22.00 |
| Wheat bran | 5.00 |
| Rapeseed meal | 3.50 |
| Soybean oil | 1.50 |
| Limestone | 6.80 |
| Dicalcium phosphate | 0.80 |
| Sodium chloride | 0.30 |
| DL-Methionine | 0.15 |
| L-Lysine hydrochloride | 0.10 |
| Vitamin–mineral premix | 0.35 |
| Total | 100.00 |

| **Nutrient** | **Content** |
| --- | --- |
| Metabolizable energy (MJ/kg) | 11.20 |
| Crude protein (%) | 17.00 |
| Crude fat (%) | 3.50 |
| Crude fiber (%) | ≤5.50 |
| Calcium (%) | 3.40 |
| Total phosphorus (%) | 0.60 |
| Available phosphorus (%) | 0.35 |
| Lysine (%) | 0.80 |
| Methionine (%) | 0.40 |
| Methionine + cystine (%) | 0.68 |
| Threonine (%) | 0.60 |
| Tryptophan (%) | 0.19 |
| Sodium chloride (%) | 0.30 |
