# Supplementary material for: Effects of heat stress on laying performance, blood hormones and biochemical indices, egg quality, and intestinal digestive enzymes in laying ducks
Source: Poult Sci. 2026 Aug 15;105(12):107617. doi: 10.1016/j.psj.2026.107617 (PMC13594849; doi:10.1016/j.psj.2026.107617)
Supplement: Supplementary file 3 [file mmc5.docx]

Table S2. The exact p-values, detailed effect sizes (Hedges' g) and 95% confidence intervals (95% CI) for all comparisons

| **index** | **p-value** | **Hedges' g** | **95% CI** |
| --- | --- | --- | --- |
| progesterone | 0.014 | 1.307 | [0.262,2.317] |
| estradiol-17β | 0.002 | 1.834 | [0.629,2.993] |
| Ovarian weight | 0.001 | 2.351 | [1.091,3.569] |
| number of hierarchical follicles | 0.001 | 3.523 | [1.954,5.052] |
| diameter of F1 | 0.006 | 1.439 | [0.413,2.431] |
| diameter of F2 | 0.001 | 1.87 | [0.79,2.914] |
| diameter of F3 | 0.001 | 1.789 | [0.725,2.818] |
| diameter of F4 | 0.002 | 1.607 | [0.577,2.604] |
| diameter of F5 | 0.198 | 0.859 | [-0.436,2.112] |
| diameter of F6 | 0.499 | 0.669 | [-1.215,2.5] |

| **index** | **p-value** | **Hedges' g** | **95% CI** |
| --- | --- | --- | --- |
| blood glucose | 0.001 | 4.932 | [3.112,6.721] |
| low‑density lipoprotein‑cholesterol | 0.741 | -0.152 | [-1.036,0.737] |
| high‑density lipoprotein‑cholesterol | 0.006 | 1.433 | [0.403,2.429] |
| triglycerides | 0.219 | 0.575 | [-0.336,1.468] |
| total cholesterol | 0.042 | 0.997 | [0.034,1.933] |
| uric acid level | 0.015 | -1.416 | [-2.523,-0.264] |
| total protein | 0.011 | 1.278 | [0.328,2.2] |
| creatinine | 0.087 | -0.081 | [-0.984,0.825] |
| albumin | 0.005 | 1.362 | [0.399,2.296] |
| lactate dehydrogenase | 0.04 | -1.256 | [-2.409,-0.055] |
| aspartate aminotransferase | 0.026 | -1.436 | [-2.657,-0.166] |
| alanine aminotransferase | 0.841 | 0.1 | [-0.858,1.053] |

| **index** | **p-value** | **Hedges' g** | **95% CI** |
| --- | --- | --- | --- |
| duodenum(maltase) | 0.022 | 2.366 | [0.292,4.349] |
| jejunum(maltase) | 0.002 | 4.462 | [1.229,7.673] |
| ileum(maltase) | 0.006 | 3.519 | [0.829,6.155] |
| cecum(maltase) | 0.283 | 0.808 | [-0.619,2.154] |
| duodenum(sucrase) | 0.011 | 2.931 | [0.563,5.224] |
| jejunum(sucrase) | 0.011 | 2.946 | [0.571,5.248] |
| ileum(sucrase) | 0.004 | 3.854 | [0.974,6.691] |
| cecum(sucrase) | 0.561 | -0.413 | [-1.696,0.917] |
| duodenum(alkaline phosphatase) | 0.003 | 4.091 | [1.074,7.073] |
| jejunum(alkaline phosphatase) | 0.009 | 3.078 | [0.631,5.456] |
| ileum(alkaline phosphatase) | 0.004 | 4.015 | [1.042,6.951] |
| cecum(alkaline phosphatase) | 0.317 | 0.744 | [-0.665,2.076] |
| duodenum(protease) | 0.006 | 3.512 | [0.826,6.145] |
| jejunum(protease) | 0.009 | 3.079 | [0.632,5.458] |
| ileum(protease) | 0.005 | 3.676 | [0.897,6.406] |
| cecum(protease) | 0.234 | 0.913 | [-0.546,2.285] |
| duodenum(lipase) | 0.017 | 2.567 | [0.391,4.658] |
| jejunum(lipase) | 0.043 | 1.906 | [0.053,3.658] |
| ileum(lipase) | 0.036 | 2.015 | [0.111,3.82] |
| cecum(lipase) | 0.207 | 0.98 | [-0.501,2.369] |
| duodenum(amylase) | 0.002 | 4.989 | [1.443,8.53] |
| jejunum(amylase) | 0.004 | 3.802 | [0.951,6.607] |
| ileum(amylase) | 0.018 | 2.532 | [0.373,4.603] |
| cecum(amylase) | 0.692 | -0.277 | [-1.551,1.029] |

| **index** | **p-value** | **Hedges' g** | **95% CI** |
| --- | --- | --- | --- |
| superoxide dismutase | 0.002 | 4.598 | [1.285,7.894] |
| malondialdehyde | 0.001 | -8.655 | [-14.564,-2.837] |
| catalase | 0.006 | 3.428 | [0.789,6.01] |
| carnitine O-octanoyltransferase | 0.017 | -2.564 | [-4.654,-0.389] |
| heat shock protein | 0.007 | -3.277 | [-5.77,-0.721] |
| glutathione peroxidase | 0.001 | 5.798 | [1.762,9.853] |
